# Comparing the knowledge, attitude and practices of health care workers in public and private primary care facilities in Lagos State on Ebola virus disease

**DOI:** 10.11694/pamj.supp.2015.22.1.6655

**Published:** 2015-10-11

**Authors:** Bilqisu Jibril Idris, Victor Inem, Mobolanle Balogun

**Affiliations:** 1 Department of Community Health and Primary Care, College of Medicine of the University of Lagos, Nigeria

**Keywords:** EVD, HCW, Primary care, Primary health care, knowledge, attitude, practice, Nigeria

## Abstract

**Introduction:**

The West African sub-region is currently witnessing an outbreak of EVD that began in December 2013. The first case in Nigeria was diagnosed in Lagos, at a private medical facility in July 2014. Health care workers are known amplifiers of the disease. The study aimed to determine and compare EVD knowledge, attitude and practices among HCWs in public and private primary care facilities in Lagos, Nigeria.

**Methods:**

This was a comparative cross-sectional study. Seventeen public and private primary care facilities were selected from the 3 senatorial districts that make up Lagos State. 388 respondents from these facilities were selected at random and interviewed using a structured questionnaire.

**Results:**

Proportion of respondents with good knowledge and practice among public HCWs was 98.5% and 93.8%; and among private HCW, 95.9% and 89.7%. Proportion of respondents with positive attitude was 67% (public) and 72.7% (private). Overall, there were no statistically significant differences between the knowledge, attitude and preventive practices of public HCWs and that of private HCWs, (p≤0.05).

**Conclusion:**

Timely and intense social mobilization and awareness campaigns are the best tools to educate all segments of the community about public health emergencies. There exists significant surmountable gaps in EVD knowledge, negative attitude and sub-standard preventive practices that can be eliminated through continued training of HCW and provision of adequate material resources.

## Introduction

The World Health Organization (WHO) defines EVD as a severe, often fatal illness in humans. It is caused by Ebola virus belonging to the filoviridae family of hemorrhagic fever viruses. The virus is transmitted from wild animals and then spreads within the human population through human to human transmission [1]. Health Care Workers (HCW) are amongst the most susceptible cohort to acquire Ebola Virus disease in the course of their duties. The statistics confirming Health Care Workers death in the current Ebola Virus Disease epidemic is staggering. This statistic is significant because of background imbalance which exists in ratio of patients to skilled/trained medical staff in Africa. EVD is infamous for its high case fatality proportion and the ease with which it spreads among contacts of infected persons [2]. Since the discovery in 1976 of Ebola virus in Sudan and Zaire, the Sudan strain has caused 6 further epidemics in man and the Zaire strain 17 further epidemics. The 2014 epidemic is the first in the West African (WA) sub region and efforts to control the epidemic has lasted more than one year. Hitherto, only Eastern and Central Africa have recorded outbreaks. The 2014 EVD epidemic in WA is the largest outbreak of the disease ever recorded [3]. On the 20th of July 2014, an acutely ill traveler from Liberia arrived in Lagos, Nigeria by air. While on admission in a private hospital, blood specimen examined at Lagos University Teaching Hospital (LUTH) returned positive for acute Ebola virus infection. This index case potentially exposed 72 other persons (at the airport and hospital) to EVD and died 5 days later [4]. By the 24th of September, 2 months after the death of the index case, 19 laboratory cases had been confirmed and 894 contacts identified and followed [4]. By the 20th of October 2014, WHO declared Nigeria free of EVD transmission. During the 3 months that the nation held its breath, 8 deaths were recorded and 11 patients recovered, indicating a case fatality ratio (CFR) of 40% [5]. There is no evidence that any research has been carried out to determine and compare the knowledge, attitude and practices (KAP) of HCW in public and private primary care facilities regarding EVD in Nigeria and ironically in Africa. As at 24th December 2014, the virus had infected over 19,431 people in WA and 7,588 people have died from the disease [6]. Interestingly, the duration and length of travel hours across continents has become significantly shorter than the incubation period of many deadly pathogens. This epidemic and many more therefore remain a global threat to health security. In addition, the most severely affected countries have very weak health systems and lack human and infrastructural resources [3]. The latter sustain disease, poverty, ignorance and fear-this recipe can cripple any economy. Nigeria is the most populated country in Africa, the world's fourth largest oil producer, and second largest supplier of natural gas. Lagos State is home to the busiest international airport in the country, a densely populated state, a port city and a haven of intense commercial activity. Outbreaks in countries like Nigeria can have a ripple effect and dampen economic outlook worldwide. Understanding and responding to the ecological, social and economic conditions facilitating disease emergence and transmission is therefore one of the major challenges of human kind today. The Nigerian experience demonstrates the need for African and indeed all developing nations to rapidly assess their readiness to manage and contain diseases like Ebola. Our overburdened infrastructure cannot be stretched further by debilitating and fatal epidemics. Periodic researches like knowledge, attitude, and practice studies can contribute to the understanding alluded to above. KAP studies are highly focused evaluations that estimate changes in human knowledge, attitude and practices in response to specific intervention, demonstration, education or circumstances [7]. The studies are attractive due to characteristics such as quantifiable data, concise presentation of results, generalizability of small sample results to a wider population and cross cultural comparability. Selection of the study population for this project is deliberate - this group of workers are the frontline responders to medical emergencies in rural and urban settings alike. This study was designed to determine and compare what two subgroups of the health community know, what their beliefs are, and what their current practices are with regards to EVD.

## Methods

**Study area:** this study was conducted in Lagos State in November 2014. Lagos shares an international boundary with the Republic of Benin. It is home to the busiest airport in Nigeria, the most densely populated of the six states that make up Nigeria's South West geopolitical zone and it is a port city. The state is divided into 3 senatorial districts.

**Sampling, sample size and data collection:** the design was a comparative cross-sectional study. Minimum sample size was obtained using the formula for comparison of proportions [8]. A total of 388 HCWs randomly picked from 17 public and private primary care facilities selected by simple ballot were interviewed. The local government areas from which the primary care centers were initially chosen were also selected by simple random sampling from the 56 LGAs and LCDAs that make up the 3 senatorial districts. Two trained health volunteers collected data using a structured 40-item questionnaire. The questionnaire covered general demographic details of respondents, knowledge and awareness of EVD, attitude/behavior questions and EVD preventive practice questions.

**Ethical consideration:** study proposal was approved by the Research and Ethics Committee of the Lagos University Teaching Hospital (LUTH). All respondents completed a consent form prior to administration of questionnaire.

**Data analysis:** data collected was processed using the statistical package for social sciences (version 21) software. Chi-squared test was used to evaluate significant differences with level of significance pre-set at p-value ≤0.05. Fisher exact test was used where appropriate. Level of knowledge was determined by a scoring system developed by the researchers. Twenty (20) questions on EVD knowledge attracted 1 mark for each correct response, and no mark for a wrong response; 10 marks and above signified good knowledge while below 10 marks was poor knowledge. Ten attitude and practice questions carried a maximum of 1 mark for each correct response. Five (5) marks and above was classified as positive attitude and good practice respectively and a score of less than 5marks represented negative attitude and poor practice respectively.

## Results

The modal age group of respondents in both public and private health facilities was 30-39 years and the mean age was 32.62 years, with a standard deviation of 5.95. There was a preponderance of female respondents across board 70.1% public, and 71.1% private, more than 85% had contact with patients (Table 1).

**Table 1:** Socio-demographic characteristics Respondents

Variable	Public (n = 194)		Private (n= 194)		χ^2^	df	pvalue	Fisher exact p
	Frequency	Percent	Frequency	Percent				
**Age(year)**								
20 – 29	55	28.4	49	25.3	14.587	3	0.002	
30 – 39	109	56.2	87	44.8				
40 – 49	25	12.9	40	20.6				
≥ 50	1	0.5	11	5.7				
Mean age	32.62 ± 5.95		34.59 ± 8.18					
**Sex**								
Male	58	29.9	56	28.9	28.928.9	1	0.824	
Female	136	70.1	138	71.1				
Education								
No formal	2	1.0	0	0	12.83	3	0.005	0.001*
Primary	2	1.0	1	0.5				
Secondary	9	4.6	28	14.4				
Tertiary	181	93.3	165	85.1				
**Duration of work (year)**								
< 1	36	18.6	49	25.3	27.685	3	< 0.001*	
1 – 5	146	75.3	103	53.1				
6 – 10	10	5.2	25	12.9				
> 10	2	1.0	17	8.8				
**Primary duty**								
Clinical services	60	30.9	107	55.2	25.399	2	< 0.001*	
Non-clinical services	99	51.0	56	28.9				
Others	35	18.0	31	16.0				
**Contact with patients**								
Yes	167	86.1	176	90.7	2.036	1	0.154	
No	27	13.9	18	9.3				

* Statistically significant

More than 75% of public respondents knew that eating bush meat could result in EVD while only 60% of private respondents correctly identified this link. Over 16% of public respondents did not know EVD could be transmitted through unprotected sex; and upto 32% of the private respondent did not know this as well. More than 85% of public and private respondent stated that drinking/bathing with salt water does not prevent EVD however, 25.3% of public and 30.4% of private respondents opined that one can tell by looking if someone has EVD; this was statistically significant (p = 0.026). Signs and symptoms of Ebola Virus Disease were correctly identified by public and private respondents except muscle pain which 7.2% public and 21.6% private were not sure of, while 3.1% public along with 3.6% of private respondents wrongly identified as false, this was statistically significant, p≤0.001% (Table 2).

**Table 2:** Distribution of Respondents by their knowledge of EVD

Knowledge of Ebola	Public (%) n = 194			Private (%) n = 194			χ^2^	p-value
	True	False	Not sure	True	False	Not sure		
**A person can be infected with Ebola if**:								
Eats bush meat	152 (78.4)	29 (14.9)	13 (6.7)	117 (60.3)	43 (22.2)	34 (17.5)	16.659	<0.001*
Touches infectedanimal	178 (91.8)	7 (3.6)	9 (4.6)	180 (92.8)	4 (2.1)	10 (5.2)	0.882	0.643
Touches aninfected person(s)	188 (96.9)	1 (0.5)	5 (2.6)	183 (94.3)	1 (0.5)	10 (5.2)	1.734	0.420
Touches a deadbody from Ebola	187 (96.4)	2 (1.0)	5 (2.6)	183 (94.3)	2 (1.0)	9 (4.6)	1.186	0.553
Having unprotected sex	139 (71.6)	32 (16.5)	23 (11.9)	101 (52.1)	66 (34.0)	27 (13.9)	18.133	<0.001*
Touches body fluids	186 (95.6)	1 (0.5)	7 (3.6)	177 (91.2)	9 (4.6)	8 (4.1)	6.690	0.035*
**A person can reduce the risk by**:								
Avoiding bush meat	173 (89.2)	10 (5.2)	11 (5.7)	169 (87.1)	8 (4.1)	17 (8.8)	1.555	0.460
Avoiding infectedanimals	186 (95.9)	2 (1.0)	6 (3.1)	184 (94.8)	3 91.5)	7 (3.6)	0.288	0.866
Using PersonalProtectionEquipment	190 (97.9)	1 (0.5)	3 91.5)	182 (93.8)	5 (2.6)	7 (3.6)	4.439	0.109
Washing handsregularly	191 (98.5)	0 (0)	3 (1.5)	188 (96.9)	0 (0)	6 (3.1)	1.024	0.312
Can tell by looking ifsomeone has EVD	94 (25.3)	108 (55.7)	37 (19.1)	56 (30.4)	82 (42.3)	53 (27.3)	7.328	0.026*
Drinking/bathingwith salt water prevent	5 92.6)	181 (93.3)	37 (19.1)	56 (30.4)	82 (42.3)	53 (27.3)	7.328	0.026*
Can stop handwashing b/c Nigeriais free	9 (4.6)	178 (91.8)	7 (3.6)	19 (9.8)	166 (85.6)	9 (4.6)	4.240	0.120
Ebola Virus Diseasecan be treated	132 (68.0)	35 (18.0)	27 (13.9)	134 (69.1)	20 (10.3)	40 (20.6)	6.628	0.036
**Signs and symptoms of Ebola include:**								
Fever	187 (96.4)	2 (1.0)	5 (2.6)	182 993.8)	2 (1.0)	10 (5.2)	1.734	0.420
Joint pains	179 (92.3)	4 (2.1)	11 (5.7)	169 (87.1)	3 (1.5)	22 (11.3)	4.097	0.129
Vomiting	185 (95.4)	2 (1.0)	7 (3.6)	180 (82.8)	3 (1.5)	11 95.7)	1.157	0.561
Diarrhea	186 (95.9)	2 (1.0)	6 (3.1)	177 (91.2)	3 (1.5)	14 (7.2)	3.623	0.163
Bleeding	184 (94.8)	4 (2.1)	6 93.1)	181 (93.3)	1 90.5)	12 (6.2)	3.825	0.148
Muscle pain	174 (89.7)	6 (3.1)	14 (7.2)	145 (74.7)	7 (3.6)	42 (21.6)	16.713	<0.001*

* Statistically significant

A significant percentage of public (41.8%) and private (30.4%) respondents believed they were not at all likely to become infected with EVD. Attitude towards EVD patients was compassionate for 99.5% of public and 88.1% of private respondents. Overwhelming majority of respondents across public (95.4%) and private (89.7%) health care settings prefer media as source of reliable information (Table 3).

**Table 3:** Distribution of Respondents by their Attitude regarding EVD

Variable	Public (n = 194)		Private (n = 194)		χ^2^	p-value
	Frequency	Percent	Frequency	Percent		
**Think can be infected**						
Very likely	34	17.5	43	22.2	8.082	0.089
Not very likely	42	21.6	42	21.6		
Somewhat likely	31	16.0	36	18.6		
Not likely at all	81	41.8	59	30.4		
No response	6	3.1	14	7.2		
**Feeling about EVD patients: compassion**						
Yes	178	91.8	171	88.1	1.397	0.237
No	16	8.2	23	11.9		
**Feeling about EVD patients: Fear**						
Yes	92	47.7	65	33.5	7.799	0.005*
No	102	52.3	129	66.5		
**Feeling about EVD patients: no feeling**						
Yes	29	14.9	18	9.3	2.929	0.087
No	165	85.1	186	90.7		
**Like to get more information**						
Yes	191	98.5	187	96.4	1.642	0.200
No	3	1.5	7	3.6		
**Get information from media**						
Yes	185	95.4	174	89.7	4.509	0.034*
No	9	4.6	20	10.3		
**Get information from religious/traditional leaders**						
Yes	63	35.1	41	21.1	9.301	0.002*
No	161	64.9	153	78.9		
**Attitude to EVD survivals**						
I feel safe	145	74.7	107	55.2	16.621	<0.001*
I feel unsafe	45	23.2	82	42.3		
No response	4	2.1	5	2.6		
**Feel comfortable providing service for EVD patients**						
Very comfortable	50	25.8	35	18.0	18.699	0.001*
Somewhat comfortable	49	25.3	31	16.0		
Very uncomfortable	34	17.5	68	35.1		
Somewhat uncomfortable	16	8.2	12	6.2		
I am not sure	45	23.2	48	24.7		
**Will refuse to treat/help EVD patients to protect you**						
Yes	84	43.3	116	59.8	13.817	0.001*
No	106	54.6	70	36.1		
No response	4	2.1	8	4.1		

* Statistically significant

A higher proportion of public respondents (74.7%) feel safe with EVD survivors compared to 55.2% of private respondents (Table 3). As regards preventive practices, 6.7% of public respondents compared to 0.5% of private respondents do not perform hand hygiene before leaving patients area and 7.2% of public respondents as opposed to 1% of priate respondents do not perform hand hygiene after removal of gloves(Table 4).

**Table 4:** Distribution of Respondents by their Practice of EVD Prevention

PracticeEbola VirusDisease prevention	Public (%) n = 194			Private (%) n = 194			χ^2^	df	p-value	Fisherexact p
	Yes	No	Not sure	Yes	No	Not sure				
Preventionmeasures withall patients	170(87.6)	24(12.4)	-	172(88.7)	22(11.3)	-		1		
**Perform hygiene:**										
Beforetouchingpatient	161(83.0)	27(13.9)	6 (3.1)	159(82.0)	21(10.8)	14 (7.2)	3.962	2	0.138	
Before leavingpatient areaafter touching	170(87.6)	13 (6.7)	11 (5.7)	178(91.8)	1 (0.5)	15 (7.7)	11.085	2	0.004*	
After gloveremoval	171(88.1)	14 (7.2)	9 (4.6)	179(92.3)	2 (1.0)	13 (6.7)	9.910	2	0.007*	
Want allcontacts to bequarantined	174(89.7)	3 (1.5)	17 (8.8)	8 (4.1)	8 (4.1)	24(12.4)	3.896	2	0.143	
Protectivemeasures withall patients	193(99.5)	1 (0.5)	0 (0)	189 (97.4)	2 (1.0)	3 (1.5)	3.375	2	0.185	0.247

## Discussion

**Principal findings:** this study essentially found significant but surmountable gaps in knowledge, attitude and preventive practices of HCW regarding EVD.

**Strengths and weaknesses:** the study provided a thorough comparison of the differences between the KAP of public HCW and the KAP of private HCW as regards EVD. This will direct policy makers in providing distinctive resources as per the needs or challenges of each group. It is noteworthy that this study was carried out roundabout the peak of the EVD outbreak in Nigeria; at a time the media was agog with Ebola campaign themes and jingles and as such respondents may report practices that do not reflect their norm or beliefs. Another drawback of the study is that it did not examine the KAP of various cadres of HCW separately, giving room for a false overall conclusion of good KAP or vice-versa.

**Important differences and similarities in results:** although no published study comparing the KAP of public and private HCW's regarding EVD was found, a few studies detailing KAP of HCW regarding other Viral Hemorrhagic Fevers exist and findings from these studies can be reasonably compared to findings of the present study as opined by Tobin E.A et al in their publication on Lassa Fever [9]. In a study to assess the KAP of health care professionals regarding EVD in India, proportion of HCW with good knowledge was 73.6% [10]. This is consistent with this study's finding of 89.2% and 83.8% good knowledge in public and private HCWs respectively. Although India is yet to record a single case of EVD, proportion of health care workers with good knowledge of the disease is quite high. This buttresses the importance of updating knowledge and preparedness by stakeholders to confront current global health challenges in the ever shrinking inter-related continents that our world has become. In Sierra Leone, public knowledge of EVD improved from 39% to 85% after 6weeks of enlightenment and social mobilization campaigns [11]. Interestingly, present study found no significant difference between the proportion of HCW with good EVD knowledge who attended Ebola training (98.7%) and those with good knowledge that did not attend any training (96.1%). In terms of attitude, 67% of public and 72.7% of private respondents in present study reported positive attitude in contrast to 83.1% of health care professionals in India. This finding is however in congruence with other studies that reveal better attitude among employees of private establishments as opposed to Government workers. Also, 35.1% of public HCW compared to 21.1% of private HCW usually get reliable information from religious and traditional leaders, however, only 8.4% of community respondents in Sierra Leone mentioned traditional and religious leaders as reliable sources of information. More than 50% of HCW in the India EVD study did not feel safe from hospital acquired infections. Contrarily, only 39.7% (17.5% public, 22.2% private) of HCW in primary care settings in Lagos think they are very likely to become infected with EVD at work.

Majority of public (74.7%) respondents feel safe with EVD survivors while only a little above half of the private respondents (55%) feel safe with EVD survivors. Perhaps EVD training imparted positively on this variable, for it found 43.3% of public HCW as opposed to 37.6% of private HCW attended EVD training. The KAP survey in Sierra Leone found that almost 70% of community respondents will not interact with EVD survivors. More than 65% of the HCW surveyed in India were willing to admit and treat EVD patients, while only 43.8% of respondents in Lagos (25.8% public, 18.0% private) reported they would feel very comfortable providing service for EVD patients. It does seem like respondents from countries and locations where EVD had struck have a more cautious disposition than countries yet to record the disease. Present study found a higher proportion of Females with positive attitude compared to their male counterparts - latter might not be unconnected with their natural caregiver/social nursing role. Older respondents in Sierra Leone also had poorer attitude compared to younger ones. However, whereas higher education imparted positively on attitude in Lagos, it did not feature as a positive effect on attitude in another Sierra Leone study [12]. Public and private HCW overwhelmingly reported taking infection prevention measures with all patients (above 87% across board). Public and Private respondents reported performing hand hygiene before touching patients, before leaving patients area after touching, and after glove removal. (83%, 87%, and 88.1% public: 82%, 91.8%, and92.3% private). There is a statistically significant difference in the results of the last two variables, p value of 0.004 and 0.007 respectively. This is an interesting finding because proportion of respondents with good knowledge in both groups is identical. Possible explanation could be available resources. Association of practice and socio-demographic characteristics in present study revealed good practice with a higher proportion of females and older respondents; health workers with < 1 to 5 years working experience, staff whose primary duties are clinical in nature, staff that come in contact with patients and ironically, workers that have not attended training on EVD. This outcome may be due to heightened awareness of the disease secondary to the trail of morbidity and mortality the disease has left in its wake in WA.

**Meaning of the study:** this study allows us to infer thus: Even though there are no statistically significant differences between the KAP of public HCW and the KAP of private HCW regarding EVD, there exists peculiar deficiencies and strengths of each group towards which policy and managerial interventions should be directed. Future research: this could focus on the KAP of specific cadres of HCW and revisit a similar exposition (as the present study) to assess improvement or decline in KAP.

## Conclusion

The public health significance of EVD is beyond its notoriety of causing overwhelming mortality and morbidity of infected persons, but also its potential for nosocomial dissemination. This study is a pioneer study comparing the KAP of health care workers regarding EVD in primary care settings - frontline responders in outbreak situations. Preparedness and optimal response to outbreaks is a function of financial and material resources, political commitment, security, good knowledge, right attitude and appropriate practices of all members of the community, especially the HCW. HCWs quest for knowledge and training should be encouraged, met, and harnessed to ensure optimum performance and successful community counseling, enlightenment and social mobilization. Improved welfare incentives and compulsory immunisation (against diseases that can be acquired at work), should be made part and parcel of conditions of service of employees at local, state and federal levels. Health education regarding emerging and re-emerging diseases and infection prevention talks can also be incorporated into routine clinic schedules. Standard protocols should be developed and enforced to ensure uniformity of practice at primary care facilities. National and State health authorities should also routinely encourage and fund researches that expose the strength and weaknesses of the health care system so that evidence based advocacy to influence policies can be embarked upon.
